# A Dual-Technology Approach: Handheld NIR Spectrometer and CNN for *Fritillaria* spp. Quality Control

**DOI:** 10.3390/foods14111907

**Published:** 2025-05-28

**Authors:** Fengling Li, Wen Lei, Juan Li, Xiaoting Wang, Jingyu Su, Tangnuer Sahati, Xiahenazi Aierkenjiang, Ruyi Tian, Weihong Zhou, Jixiong Zhang, Jingjing Xia

**Affiliations:** 1 College of Life Science and Technology & School of Pharmaceutical Sciences and Institute of Materia Medica, Xinjiang University, Urumqi 830017, China; 2 Institute of Agro-Products Storage and Processing, Xinjiang Key Laboratory of Processing and Preservation of Agricultural Products, Xinjiang Academy of Agricultural Sciences, Urumqi 830091, China

**Keywords:** NIR, CNN, *Fritillaria* spp., visualization, geographical origin

## Abstract

*Fritillaria* spp. has an extremely high edible and medicinal value. Different parts of it exhibit significant variations in medicinal efficacy. To rapidly and accurately identify the origin and adulteration of *Fritillaria* spp., a handheld near-infrared spectrometer was combined with a convolutional neural network (CNN) to establish an efficient and convenient quality assessment method. First, for the origin of *Fritillaria* spp., the CNN could achieve high accuracy, with 100 ± 0%. The features contributing to the origin of *Fritillaria* spp. were visualized using gradient-weighted class activation mapping (Grad-CAM). For the adulteration of *Fritillaria* spp., compared with partial least squares regression (PLSR), the CNN yielded the best performance, with the R^2^ of the test set being 0.9897. Additionally, to improve the interpretability of the adulteration model, a CNN model was established using data whose dimensions had been reduced by PCA (PCA–CNN), which also achieved an R^2^ of 0.9876. The features extracted by PCA focused on 1400–1500 nm, which was consistent with Grad-CAM. The visualization of Grad-CAM and the adulteration detection model achieved mutual validation, showing the effectiveness of both methods in analyzing the samples. The experimental results demonstrated that the integration of a handheld near-infrared spectrometer with a CNN enabled both reliable authentication of *Fritillaria* spp. geographical origins and quantitative determination of adulteration levels, establishing a novel analytical framework for rapid quality evaluation of *Fritillaria* spp. materials.

## 1. Introduction

*Fritillaria* spp. is a kind of perennial herb, not only a key component in traditional Chinese medicinal (TCM) preparations but also widely used in culinary practices, such as soup preparation, congee cooking, and herbal tea infusion. *Fritillaria* spp. encompasses multiple species, including *Fritillaria cirrhosa* D. Don, *F. ussuriensis* Maxim., *F. pallidiflora* Schrenk, and *F. thunbergii* Miq. [1]. Although there are differences in their phytochemical profiles, the high degree of morphological similarity results in the prevalence of counterfeit or non-compliant samples in the market.

With the advancement of precision medicine, numerous analytical methods have been developed to distinguish the *Fritillaria* spp. For example, Liang et al. used ribosomal DNA and super barcoding of the chloroplast genome to identify the cultivation origin of *Fritillaria taipaiensis* P. Y. *Li* [2]. A new quantitative detection method was developed based on molecular markers by Liu et al., specifically using single nucleotide polymorphisms, which were capable of distinguishing *Fritillaria cirrhosa* D. Don from its adulterated species [3]. Dai et al. applied gas chromatography–mass spectrometry (GC-MS) combined with chemometrics to effectively differentiate *Fritillaria* species through volatile organic compound profiling [4]. Based on the content of the alkaloid, both Liu et al. [5] and Zhu et al. [6] employed liquid chromatography–mass spectrometry (LC-MS) to analyze the nine primary isosteroidal alkaloids and utilized PCA and hierarchical clustering analysis (HCA) to differentiate *Fritillaria cirrhosa* D. Don from *Fritillaria pallidiflora* Schrenk. The abovementioned detection methods possess high accuracy and specificity, but they have strict sample requirements, high costs, and relatively high technical difficulty. However, these methods face two primary limitations: (1) sample preparation is intricate; (2) the instruments are huge and sophisticated, which restricts the application of the methods on-site.

Near-infrared (NIR) spectroscopy covers the spectral region of 780–2526 nm, which is consistent with the overtone and combination bands of hydrogen-containing group vibrations (such as O–H, N–H, C–H) in organic molecules, enabling the acquisition of characteristic information about these functional groups in samples. When combined with chemometrics, NIR has the advantages of being non-destructive, requiring minimal pretreatment, and enabling rapid analysis, making it applicable to many areas, such as TCM quality assessment, food science, and tobacco analysis. In the origin identification of TCM, Xin et al. used mid-infrared (MIR) spectroscopy to build an origin discrimination model to distinguish the origin of wild and cultivated ginseng, which demonstrated its efficacy in sample traceability [7]. Liu et al. employed NIR and MIR spectroscopy to identify the geographical origin of Eucommiae Cortex across eight provinces, and the accuracies of KNN and PLS-DA were 100% and 98.61%, respectively [8]. NIR spectroscopic fingerprinting combined with pattern recognition was used to classify the origin of *Panax notoginseng*, and the model of soft independent modeling of class analogies (SIMCA) achieved 100% accuracy [9]. Li et al. used MIR and NIR spectroscopy with ResNet to classify different varieties of *Gastrodia elata* Bl for TCM species identification [10,11]. In their research on TCM adulteration, Coqueiro et al. employed MIR and NIR spectroscopy to detect cinnamon adulterants (coffee husks, cornmeal), and validated the method through commercial sample screening, and the results demonstrated its efficacy [12]. Amirvaresi et al. utilized NIR spectroscopy to detect four plant-derived adulterants in saffron, the coefficient of determination (R^2^) (0.95–0.99) of PLSR of which demonstrated robust spectroscopic authentication efficacy [13]. Additionally, quantitative analysis of bioactive constituents in TCM has been extensively investigated by using NIR spectroscopy. To analyze the content of bilirubin quickly, Tian et al. established a prediction model between the NIR spectroscopy and the content of bilirubin. The R^2^ could reach 0.979, indicating a good prediction ability of the model [14]. Peng et al. applied CNN and gated recurrent unit (GRU) models to analyze the correlation between NIR spectral data and the content of active ingredients in Radix Gentianae Macrophyllae, specifically loganic acid and gentiopicroside. The R^2^ values of the model prediction were over 0.97 [15].

In the research on *Fritillaria* spp., An et al. differentiated five *Fritillaria* Bulbus species and three varieties of *F. cirrhosa* using multi-technique integration (MIR, TLC imaging, and metabolomics) [16]. Zhou et al. effectively discriminated between *Fritillaria cirrhosa* D. Don and other *Fritillaria* species using KNN [17]. Wei et al. employed laser-induced breakdown spectroscopy (LIBS) to detect adulteration in *F. cirrhosa* powder, achieving an R^2^ of 0.9983 in PLSR [18]. Kabir et al. quantified polysaccharides using NIR spectroscopy [19]. Furthermore, Zhao et al. determined the content of peimin A and peimin B in *Fritillaria thunbergii* Miq. [20]. The existing research has established identification models for distinguishing *Fritillaria cirrhosa* D. Don from other species, demonstrating the potential of using NIR spectroscopy to identify *Fritillaria cirrhosa* D. Don. However, the existing research employed benchtop NIR spectrometers, which limited the market regulation. Meanwhile, the current research focused on the identification of *Fritillaria cirrhosa* D. Don, but lacked the quality evaluation of other *Fritillaria* species such as *Fritillaria thunbergii* Miq., *Fritillaria ussuriensis* Maxim., and *Fritillaria pallidiflora* Schrenk.

Therefore, in this research, a handheld near-infrared spectrometer was utilized to analyze four *Fritillaria* species. To comprehensively evaluate the applicability of the method, the study encompassed the identification of origins and the accurate prediction of adulteration content. Part I focuses on the identification of *Fritillaria* spp. origins. Three spectral preprocessing methods were systematically integrated with four machine learning algorithms to establish a robust model for determining the geographical origin of *Fritillaria* spp. Furthermore, to enhance the reliability and interpretability of the model, Monte Carlo sampling and gradient-weighted class activation mapping (Grad-CAM) were synergistically involved, with the former generating multiple datasets and the latter visualizing the important features. In Part II, we detected and analyzed the adulteration of *Fritillaria* spp. from linear and nonlinear perspectives. Contributions and key highlights: (1) first, we integrated a handheld NIR spectrometer with a CNN for the quality assessment of *Fritillaria*; (2) to enhance the ability of the model to explain chemical information, we utilized Grad-CAM to visualize the important features, thereby improving the chemical interpretability of models; (3) the combined use of a CNN and PCA for the prediction of adulteration content achieved a high R^2^ value of 0.9897. This result could offer a new technique for the quality assessment of *Fritillaria* spp.

## 2. Experimental Materials and Methods

### 2.1. Sample Preparation

Dried bulbs of four *Fritillaria* species—*Fritillaria cirrhosa* D. Don, *Fritillaria ussuriensis* Maxim., *Fritillaria pallidiflora* Schrenk, and *Fritillaria thunbergii* Miq.—were procured from Xinjiang Erdaoqiao Chinese Herbal Medicine Store, Urumqi, Xinjiang, China in 2024. The samples were identified by Researcher Jiang He of the Xinjiang Institute of Materia Medica, Urumqi, Xinjiang, China as the corresponding *Fritillaria* variety. Four batches of each kind of *Fritillaria* spp. were collected. All samples were crushed and sieved through 40 mesh sieves, labeled and stored in sealed bags.

To reflect real-world conditions and improve the applicability of the model, we selected *Fritillaria ussuriensis* Maxim. as the adulterant for *Fritillaria cirrhosa* D. Don (the two kinds of *Fritillaria* have extremely similar morphologies, but their prices differ significantly.). Additionally, the number of samples was set according to the distribution of different adulteration proportions. The adulteration proportions of *Fritillaria ussuriensis* Maxim. in *Fritillaria cirrhosa* D. Don ranged from 0.1% to 50%. Specifically, an adulterated sample was set every 0.5% within the range of 0.1% to 10%, and every 5% within the range of 10% to 50%. Detailed information is displayed in Table S1. A total of 29 adulterated samples were obtained and stored at room temperature after thorough mixing.

### 2.2. Near-Infrared Spectra

An IAS-8120 handheld near-infrared spectrometer (Xunjie Guangyuan Company, Shanghai, China), with a mass of 2 kg and dimensions of 116 mm × 263 mm × 303 mm (length × width × height), was used for NIR spectral acquisition. The spectrometer used low-power tungsten halogen lamps serving as the light source and employed an indium gallium arsenide detector. The wavelength range spans from 900 to 1700 nm, with a data interval of 1 nm. Then, about 12 g of powdered *Fritillaria* spp. was loaded into the sample tank of the handheld spectrometer to completely cover the light spot. For the identification of origins, 45 spectra were collected for each batch of *Fritillaria* spp. A total of 180 spectra were collected for each origin (4 batches × 45 acquisitions), and 720 spectra were collected in total (4 origins × 180 spectra). Aiming at adulterated samples, 20 spectra were collected for each proportion. In total, 580 spectra were obtained (29 levels × 20 replicates). All these spectra were then exported to the CSV format.

### 2.3. Algorithms

#### 2.3.1. Data Preprocessing

Spectra not only contain valuable information but also carry redundant or interfering information. Thus, to eliminate interference and enhance the potentially useful spectral information [21], several preprocessing methods based on the characteristics of the spectra and samples were employed in this study. In the data processing of this study, MATLAB (R2023a) was used throughout. Savitzky–Golay (S–G) smoothing is a convolution-based digital filtering technique that utilizes polynomial fitting to approximate localized spectral data within a sliding window. By iteratively replacing the central window value with the optimized polynomial estimate, this method simultaneously suppresses noise while preserving critical spectral features such as peak morphology and baseline characteristics [22]. Standard normal variate (SNV) transformation corrects multiplicative light scattering effects induced by particle size variations through row-wise normalization of each spectrum [23]. Derivative (Der) processing enhances spectral resolution by differentiating overlapping peaks, thereby amplifying subtle differences between adjacent spectral features. Adaptive iterative re-weighted penalized least squares (airPLS) employs penalized least squares fitting to automatically estimate and eliminate baselines, effectively handling diverse baseline complexities and ensuring accurate spectral feature representation [24]. Considering the characteristics of *Fritillaria* spp. samples and preprocessing methods, S–G smoothing was selected to eliminate the instrumental noise, and airPLS was used to remove the baseline. Furthermore, SNV transformation was applied to eliminate the light scattering caused by the particle size, and Der processing was employed to distinguish the overlapping spectral peaks.

#### 2.3.2. Machine Learning Algorithms

PCA transforms data from a high-dimensional space to a lower-dimensional space (known as principal components), achieving this by linear transformation. These components are ordered based on their variance, where the components with the highest variance are ranked first [25]. Consequently, the spatial position distribution of the samples could be viewed through the first two or three principal components. PLS-DA is based on PLS and subsequently incorporates a discriminant analysis. It is used for category discrimination. The parameters of PLS-DA are similar to those of PLSR. The goal is to find latent variables that maximize the ability to discriminate between samples of different categories [26]. Different from the dimensionality reduction ideas of PCA and PLS-DA, support vector machine (SVM) projects data into a high-dimensional space to find an optimal hyperplane. This optimal hyperplane is determined by support vectors, which are the training sample points closest to the hyperplane [27]. The decision tree (DT) simulates the decision process by constructing a tree structure for both classification and regression. On each internal node, the algorithm must select a feature to test, and the purpose of feature selection is to segment the dataset into a subset that is as pure as possible (i.e., containing as many samples of the same class as possible) [28].

#### 2.3.3. Convolutional Neural Network

CNN is a deep learning model that usually consists of an input layer, convolutional layers, pooling layers, fully connected layers, and an output layer [29]. In this study, data normalization was used to accelerate the training process and prevent overfitting. During the training process, the network adjusted the weights and biases through back-propagation in order to minimize the loss function. In this study, the architecture of CNN is summarized in Table S2. The network included five convolutional layers, with the number of filters being 16, 32, 64, 128, and 256, respectively. The size of a filter was 3 × 1. Each convolutional layer was followed by batch normalization and rectified linear unit (ReLU) activation. The training configuration adopted the Adam optimizer with an initial learning rate of 1 × 10^−4^, with the initial learning rate being reduced by 20% every 50 epochs. Mini-batches of 10 samples were used for up to 1500 epochs, and validation metrics were monitored after each training epoch to ensure stable convergence.

Grad-CAM is a gradient-based visualization method that utilizes the gradient information of a CNN to highlight the activation regions of specific categories in the input image. Specifically, Grad-CAM calculates the gradients of the category output scores with respect to the final convolutional layer and generates heatmaps. This approach thereby aids in understanding how the spatial information in the input image is relied upon for the prediction of the model [30]. In this research, on the basis of the accurate identification of *Fritillaria* spp. using a CNN, Grad-CAM was utilized to visualize the characteristic regions for each geographical origin of *Fritillaria* spp. Firstly, we calculated the average spectrum of each origin and input it into a CNN model. Secondly, we determined whether the origin predicted by the CNN model was consistent with the true origin. Thirdly, if the origin predicted by the CNN model was consistent with the true origin, we calculated the class of the output layer and the output feature map information of the last convolutional layer. Then, we weighted each channel of the feature map in the convolutional layer by the gradient of the class with respect to that channel. Through backpropagation, the CNN model identified key feature peak locations for each class. It is essential to note that the reliability of the visualized information depended on the accuracy of the CNN prediction of the model. If the CNN model made accurate predictions, the visualized information could be considered reliable. Conversely, inaccurate predictions made by the CNN model indicated significant errors in the visualization.

#### 2.3.4. Data Segmentation

For the identification of *Fritillaria* spp. origins, 4 batches were collected from each origin, with 45 spectra collected from each batch. Monte Carlo sampling was used to randomly select one-third of the samples as the test set (60 samples), and the remaining samples as the training set (120 samples). In the adulteration model, 20 spectra were collected for each adulteration proportion. These spectra were then divided into a training set and a test set at a ratio of 1:2, resulting in 13 spectra for the training set and 7 spectra for the test set, respectively.

### 2.4. Model Effect Evaluation

The confusion matrix is commonly used to assess the performance of a classification model. In the confusion matrix, each row represents the actual categories, while each column represents the predicted categories. The diagonal elements of the confusion matrix indicate that the samples are predicted correctly. In addition, the confusion matrix can provide several metrics of the model, such as accuracy, precision, recall, and F1-score [31].

The formula for *accuracy* is Equation (1):

(1) $$Accuracy=\frac{\left(TP+TN\right)}{\left(TP+TN+FP+FN\right)}$$

The formula for *precision* is Equation (2):

(2) $$Precision=\frac{TP}{\left(TP+FP\right)}$$

The formula for *recall* is Equation (3):

(3) $$Recall=\frac{TP}{\;(TP+FN)}$$

The formula for *F*1*-score* is Equation (4):

(4) $$F1Score=\frac{2TP}{\left(2TP+FP+FN\right)}$$

*TP* stands for true positives, which refers to the samples that are positive both in model predictions and true values. *TN* stands for true negatives, which refers to the number of samples whose model predictions and true values are negative. *FP* stands for false positives, which represents the number of samples whose model predictions are positive but true values are negative. *FN* stands for false negatives, which indicates the number of samples whose model predictions are negative but true values are positive.

In a regression model, the coefficient of determination (*R*^2^) is commonly used as a metric to evaluate the performance of the model. Generally, an *R*^2^ value close to 1 indicates that the model is well-fitted, while an *R*^2^ value of 0 indicates that the model is poorly fitted. The formula for *R*^2^ is expressed as Equation (5):

(5) $$R^{2}=1-\frac{\sum_{i=1}^{n}{\left(y_{i}-{\hat{y}}_{i}\right)}^{2}}{\sum_{i=1}^{n}{\left(y_{i}-{\bar{y}}_{i}\right)}^{2}}$$

Root mean square error (*RMSE*) is one of the commonly used metrics in regression modeling to measure the performance of predictive models. *RMSE* is calculated as the square root of the average of the squared deviations between the true values and the predicted values. Based on the training set and the test set, the *RMSE* can be divided into root mean square error of cross-validation (RMSECV) and root mean square error of prediction (RMSEP). The smaller the values of the RMSECV and the RMSEP, the smaller the total error between the predicted value and the true value. The formula for calculating *RMSE* is expressed as Equation (6):

(6) $$RMSE=\sqrt{\frac{1}{n}\sum_{i=1}^{n}{\left(y_{i}-{\hat{y}}_{i}\right)}^{2}}$$

where n is the number of samples, $y_{i}$ represents the true observation, ${\bar{y}}_{i}$ is the average of the true observations, ${\hat{y}}_{i}$ represents the predicted value.

## 3. Results and Analysis

### 3.1. Near-Infrared Spectroscopy and Data Preprocessing

**Figure 1:**
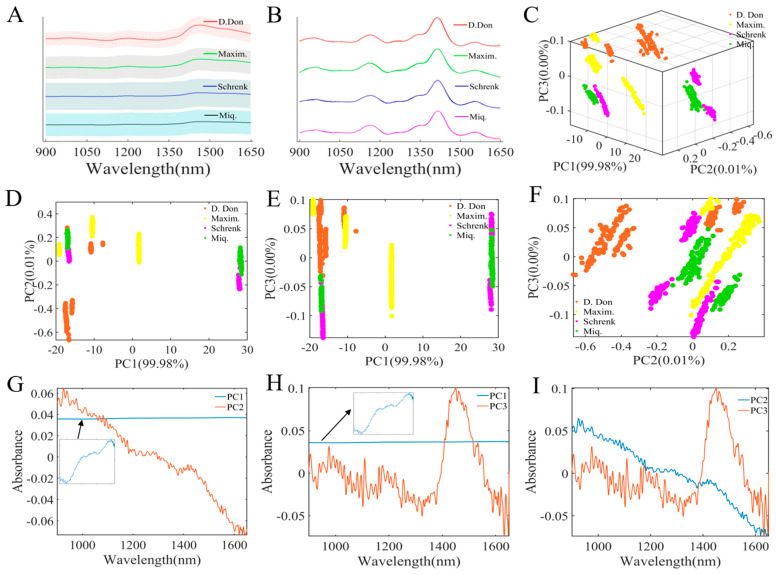
(**A**) Average NIR spectra. (**B**) Average NIR spectra after applying second-order derivatives. (**C**) Scores of the first three principal components. (**D**–**F**) Scores of different principal component pairs: (**D**) PC1 and PC2, (**E**) PC1 and PC3, (**F**) PC2 and PC3. (**G**–**I**) Loading of different principal component pairs: (**G**) PC1 and PC2, (**H**) PC1 and PC3, (**I**) PC2 and PC3.

The NIR spectra of four species of *Fritillaria* spp. are displayed in Figure 1A. Although the spectral peak positions are very similar, the intensities of the spectra have significant differences, such as at 1465 nm. Moreover, the solid lines represent the average spectrum, while the shaded areas reflect its standard deviation, demonstrating that there are significant differences in the spectra of each origin. In order to eliminate the differences within the region and maximize the differences between regions, preprocessing methods were adopted to process the spectra. Figure S1A shows the spectrum after S–G + SNV preprocessing, with the smoothing window size set to 51 to effectively suppress noise. After that, combined with the SNV, it successfully eliminated the difference within each region, specifically reflected in the narrowing of the shaded areas in each region. In order to maximize the differences between the obvious regions, the derivative was utilized to differentiate overlapping peaks by employing a first-order derivative cubic polynomial. As can be seen from Figure 1B, the spectral peaks became more prominent. In addition, Figure S1B demonstrates the spectrum after baseline correction by using the airPLS algorithm. Compared with Figure 1A, airPLS effectively eliminated baseline interference, resulting in a lower standard deviation of the original data than that of untreated samples. However, a comparison with Figure S1A shows that the standard deviation of spectra still existed. Based on the preprocessing, the standard deviation of spectra could be significantly reduced, the baseline drift could be effectively eliminated, and the identification of peaks could be enhanced. However, it remained extremely difficult to identify the four origins of *Fritillaria* spp. through NIR fingerprinting.

### 3.2. Principal Component Analysis

Due to the high similarity among the NIR spectra of the four *Fritillaria* spp. origins, it was ineffective to differentiate them solely based on the spectra. PCA concentrates the main information of the spectra into a few dimensions through data dimensionality reduction, facilitating the visualization of the spatial distribution of the sample points. Figure 1C–F presents the sample distributions in two and three dimensions, respectively. The horizontal, vertical, and z-axis coordinates are the first principal component (PC1), the second principal component (PC2), and the third principal component (PC3), with the contributions accounting for 99.98%, 0.01%, and 0.00% of the total variance, respectively. In Figure 1D, *Fritillaria pallidiflora* Schrenk and *Fritillaria thunbergii* Miq. cluster together and are nearly indistinguishable, and some samples of them overlap with *Fritillaria cirrhosa* D. Don. However, *Fritillaria cirrhosa* D. Don and *Fritillaria ussuriensis* Maxim. can be distinguished based on their positions in the first two principal component (PC) dimensions. Figure 1G–I displays the loading plots of the first three principal components, where the information extracted by PC1 reflects features similar to those in the original spectra of *Fritillaria* spp., while the loading plot of PC2 is almost entirely opposite to the loading plot of PC1. Among the information from PC1 and PC3 (Figure 1E,H), the most notable observation is that one batch of *Fritillaria ussuriensis* Maxim. is clearly distinguished from the other *Fritillaria* species. PC3 primarily exhibits a characteristic peak at 1460 nm, which indicates that this spectral feature plays a significant role in distinguishing *Fritillaria ussuriensis* Maxim. Based on the features represented by PC2 and PC3, *Fritillaria cirrhosa* D. Don can be clearly distinguished from other *Fritillaria* samples, primarily corresponding to a spectral feature at 1460 nm, which contributes to its distinction. In summary, 1460 nm is the key wavelength for differentiating *Fritillaria cirrhosa* D. Don and *Fritillaria ussuriensis* Maxim. Meanwhile, *Fritillaria* spp. samples from the same origin but different batches are located in different positions, indicating that the differences between batches are relatively significant, potentially influenced by factors such as harvesting cycles and storage duration. Subsequent research can employ digital solutions to predict the harvesting cycles and storage times of TCM, thereby providing intelligent strategies for precise medication administration.

PCA has the potential to differentiate *Fritillaria* spp. from four different geographical origins, which fully demonstrates the feasibility of using handheld near-infrared spectrometers for tracing the origin of *Fritillaria* spp. Subsequently, machine learning algorithms were employed to establish identification models for *Fritillaria* spp. origins to verify the accuracy and reliability of origin identification.

### 3.3. The Identification Model of Fritillaria *spp.* Origin

**Figure 2:**
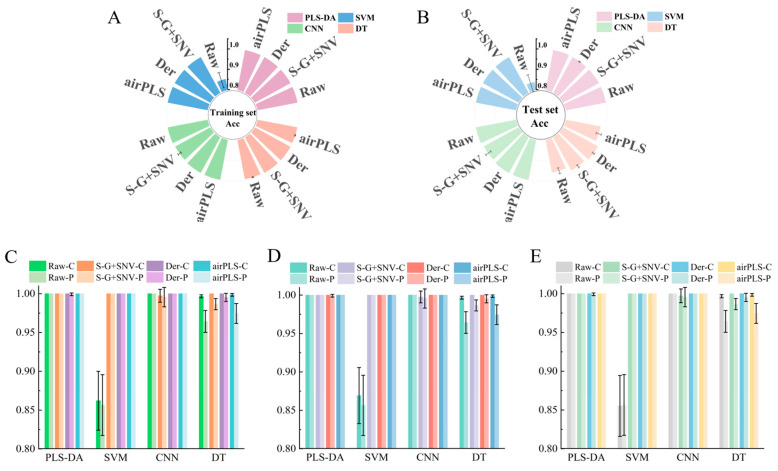
(**A**,**B**) Accuracy of the training set and the test set, respectively. (**C**–**E**) F1-score, precision, and recall, respectively (the letter “C” stands for the “training set”, while the letter “P” stands for the “test set”).

Four kinds of algorithms were employed to construct the identification model of the *Fritillaria* spp. origin. The parameters of the model were tuned using 10-fold cross-validation. In order to enhance the robustness of model performance, we employed Monte Carlo sampling to generate 20 groups of datasets, and then we established a predictive model based on these generated datasets. The evaluation of the model was based on the average (ave) and standard deviation (std) of four metrics (accuracy, F1-score, precision, and recall). The model accuracy for the training set (shown in Figure 2A) and the test set (shown in Figure 2B) is presented, with detailed information displayed in Table S3. Specifically, the PLS-DA model demonstrated excellent performance. For most cases, the classification accuracy was 100%, with no variation (±0%). However, for the test set results obtained after derivation preprocessing, the accuracy was 99.92% ± 0.19%. The SVM model achieved an accuracy of 84.02 ± 4.25% on the raw test set data. However, when three preprocessing methods were applied, the results for all other cases improved to 100 ± 0%. For the DT model, the performance after all three preprocessing methods (98.63 ± 0.94%, 99.50 ± 0.55%, and 97.42 ± 1.31%) was better than the performance using the raw spectral data (96.33 ± 1.48%). In the case of the CNN, all the results were 100 ± 0%, except for the model result of 99.48 ± 1.55% obtained after SG + SNV processing. We can see from the above results that different algorithms combined with different preprocessing methods yield different results. For example, after applying preprocessing, the model performance of SVM and DT improved significantly. However, for PLS-DA and the CNN, certain preprocessing steps led to a degradation in model performance. In addition, except for the SVM model performance on the raw data, which was 84.02 ± 4.25%, all the other models achieved a performance higher than 96.33 ± 1.48%. Moreover, for three metrics, F1-score (Figure 2C), precision (Figure 2D), and recall (Figure 2E), detailed information is presented in Tables S4–S6. These metrics show the same trend as accuracy. Collectively, this indicates that the combination of handheld near-infrared spectrometers and machine learning algorithms could effectively distinguish the different origins of *Fritillaria* spp.

### 3.4. Feature Visualization Analysis

**Figure 3:**
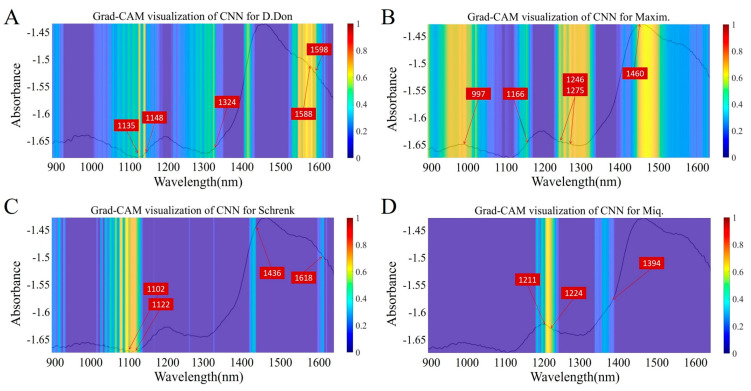
Grad-CAM visualization of the CNN for (**A**) *Fritillaria cirrhosa* D. Don, (**B**) *Fritillaria ussuriensis* Maxim., (**C**) *Fritillaria pallidiflora* Schrenk, and (**D**) *Fritillaria thunbergii* Miq. The numbers in the plots indicate the selected wavelengths.

To explore the primary feature differences between four kinds of *Fritillaria* spp., Grad-CAM was employed to visualize the feature regions following accurate identification using the constructed CNN model. For each region, we used the average spectra as the representative spectra and input the average spectra into the constructed CNN model. In this case, if the prediction of the CNN model was consistent with the actual origin, Grad-CAM was employed to visualize the important features. The visualized results of Grad-CAM for four different origins of *Fritillaria* spp. are shown in Figure 3. Different colors are assigned to regions in the Grad-CAM visualized results based on their importance. More important regions are displayed as yellow bands, while less important ones are shown as blue bands. In order to verify the effectiveness of Grad-CAM visualization, the spectral data of *Fritillaria* spp. were combined and then analyzed. For example, for *Fritillaria cirrhosa* D. Don, the visualized regions in the Grad-CAM results are concentrated in the 1550–1600, 1324, and 1100–1200 nm (Figure 3A), corresponding to the chemical information of a C–H stretching and bending vibration, a C–H or N–H stretching vibration, and an O–H and C–H stretching vibration (usually related to water or substances containing -OH functional groups, especially sugars, fatty acids, and other components), respectively. According to the report by Chen et al. [32], the polysaccharides of *Fritillaria cirrhosa* D. Don exhibited unique characteristics that aligned well with the visualized information. Regarding *Fritillaria ussuriensis* Maxim., several visualized regions are shown (Figure 3B). For instance, the wavelength around 1460 nm indicates the information of a C–H stretching vibration, which is commonly found in organic molecules, especially fatty acids and fatty compounds. The C–H stretching or bending vibrations are observed at 997 and 1246 nm, and these vibrations are mainly present in fatty acids, alkaloids, or other hydrocarbon-containing components. The C–H and O–H stretching vibrations corresponding to 1166 nm and 1275 nm might indicate the presence of moisture, some alkaloids, or sugars in *Fritillaria* spp. Unlike *Fritillaria cirrhosa* D. Don*,* the visualized regions in *Fritillaria ussuriensis* Maxim. are associated with the presence of alkaloids. Studies have demonstrated that *Fritillaria ussuriensis* Maxim. contains the highest alkaloid content among the related species [33]. This high alkaloid content is believed to be a key factor contributing to its unique medicinal value. The characteristic visualized regions of *Fritillaria pallidiflora* Schrenk are mainly focused around 1100 nm (Figure 3C). This wavelength range corresponds to the O–H stretching vibration, which is typically associated with plant components containing water or -OH functional groups, such as sugars and organic acids. As shown in Figure 3D, for *Fritillaria thunbergii* Miq., the substances corresponding to the visualized region are mainly composed of substances found in fatty acids, alkaloids, or other components containing hydrocarbons, with the visualized region concentrated around 1200 nm. This characteristic information, which includes polysaccharides and alkaloids of *Fritillaria thunbergii* Miq., is also consistent with [34], and the results validate the interpretability and effectiveness of the visualization area.

It can be concluded that the combination of a handheld near-infrared spectrometer and machine learning algorithms can achieve high-precision identification of the geographical origin of *Fritillaria* spp. Based on the accurate identification of the geographical origins of *Fritillaria* spp. achieved using a CNN, Grad-CAM was applied to visualize the spectral feature regions of each *Fritillaria* spp. By referencing the existing literature reports, the interpretability and effectiveness of these visualized spectral feature regions, which are crucial for accurately distinguishing different *Fritillaria* spp. and thus ensuring product quality, were validated. This validation provides new technical support and solutions for the subsequent quality control of *Fritillaria* spp.

### 3.5. Adulteration Prediction Model of Fritillaria cirrhosa *D. Don*

**Figure 4:**
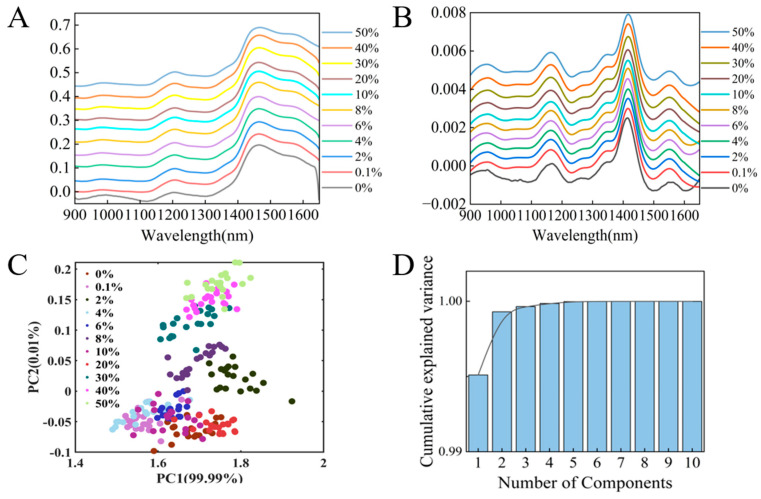
(**A**) Raw adulteration spectrum. (**B**) Adulteration spectrum after preprocessing with second-order derivatives. (**C**) Scores of the first two PCs. (**D**) Cumulative variance explained by different numbers of PCs.

To further broaden the application scope of the research methods, a handheld near-infrared spectrometer combined with multiple regression algorithms was utilized to establish the prediction model. According to the prevalence of adulteration in the market, *Fritillaria ussuriensis* Maxim. was chosen as the adulterant to be blended into *Fritillaria cirrhosa* D. Don. The NIR spectra at varying adulteration ratios are illustrated in Figure 4A, while Figure 4B presents the adulterated spectra following second derivative preprocessing. To maintain clarity in data presentation, selected NIR spectra with key representative data points are shown. Since including all spectra would result in excessive complexity in the figure, only the most relevant data points are plotted, thus allowing for a clear focus on the key trends. Both in the raw spectra and the spectra after the second derivative, there was no visually discernible change in the absorption peaks as the concentration of adulterants increased. Therefore, multiple regression algorithms were employed next for the analysis of adulteration in *Fritillaria* spp. In order to improve the predictive performance of the model, regression prediction models were built using two types of data: full-spectrum and data after PCA, as well as two types of algorithms: linear (PLSR) and nonlinear (CNN). Finally, these four data models were analyzed and compared to comprehensively evaluate the model effects. The PCA scatter plot of the spectra is illustrated in Figure 4C, wherein PC1 and PC2 accounted for 99.93% of the total variance (PC1 = 99.51%, PC2 = 0.42%). The first ten principal components of the spiked samples were extracted, with their cumulative explained variance depicted in Figure 4D.

#### 3.5.1. PLSR Regression Model

**Figure 5:**
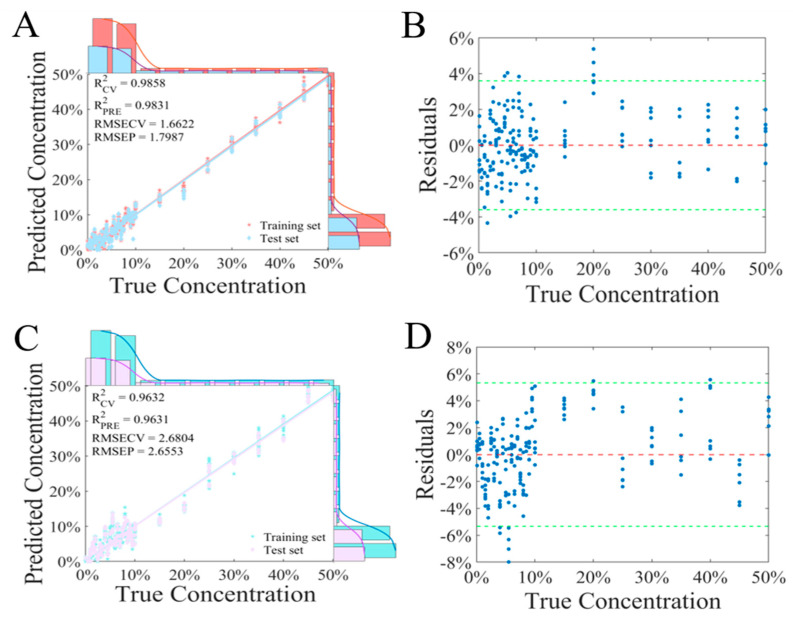
(**A**) Fitting plot of PLSR for the raw spectra. (**B**) Residual scatter plot of PLSR. (**C**) Fitting plot of PLSR for the spectra treated with PCA. (**D**) Residual scatter plot of PCA-PLSR.

A prediction model for *Fritillaria* spp. adulteration was established using a combination of three preprocessing methods with PLSR. For the raw spectra (Figure 5A), the R^2^ of the training set and the test set was 0.9858 and 0.9831, and the RMSECV and the RMSEP were 1.6622% and 1.7987%, respectively. From the residual scatter plot (Figure 5B), it can be seen that the prediction error was basically within 4%. For the preprocessing methods of S–G + SNV, Der, and airPLS, the R^2^ of the test sets was 0.9807, 0.9806, and 0.9826, respectively. Compared to the raw data, the preprocessed data did not improve the prediction performance of the model. Instead, the prediction performance of the model decreased. When combined with PCA (using the first 10 components), the performance and the residual scatter plot of the model are displayed in Figure 5C,D. The R^2^ of the test set for PCA-PLSR was 0.9631. Although there were some differences in the performance of the models built using different ways, the R^2^ values of the models were all above 0.98 (except for PCA-PLSR), indicating that a handheld near-infrared spectrometer combined with the PLSR algorithm could be used for predictive analysis of *Fritillaria* spp. adulteration.

#### 3.5.2. CNN Regression Model

**Figure 6:**
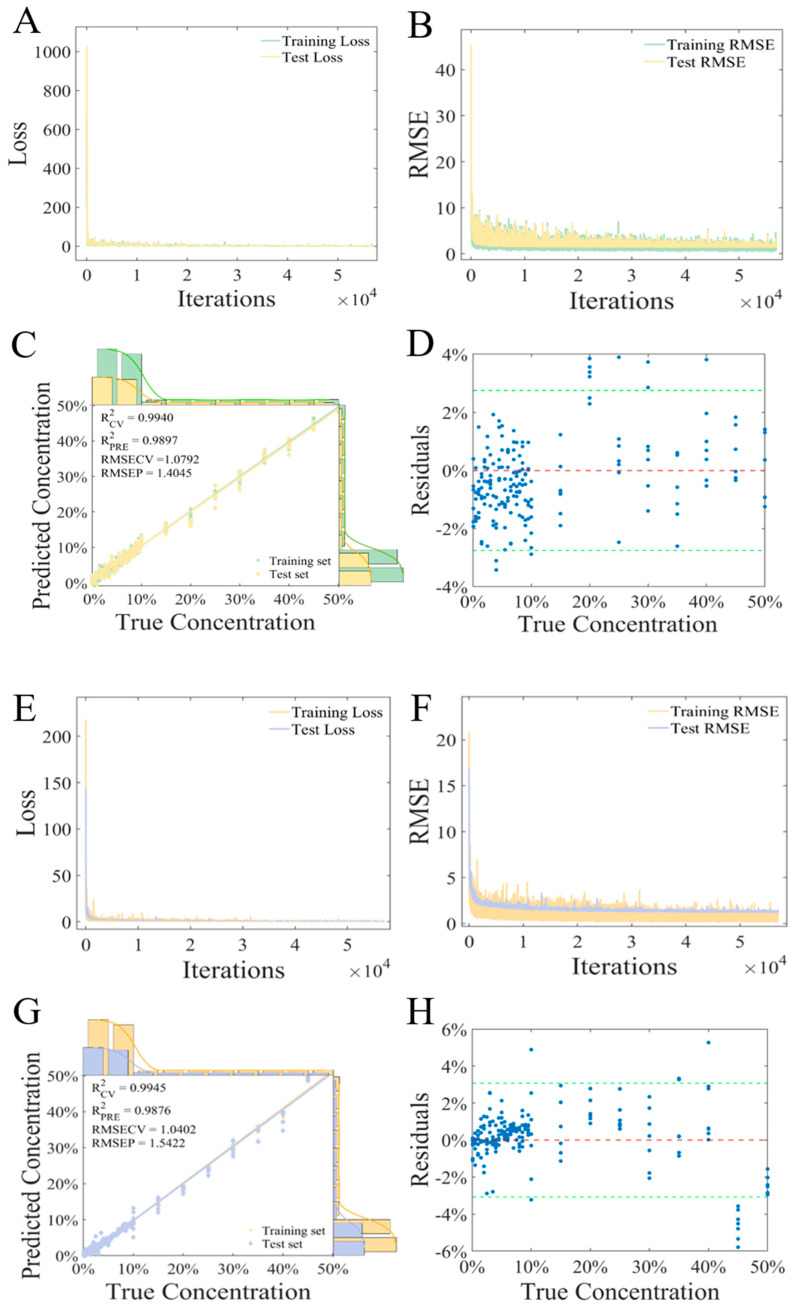
(**A**–**D**) Loss value, RMSE, regression model, and residual scatter plot of the CNN model after airPLS, respectively. (**E**–**H**) Loss value, RMSE, regression model, and residual scatter plot of the PCA–CNN model, respectively.

Similarly, three preprocessing techniques were utilized to optimize spectral data, and the processed data were input into the CNN model to establish a regression prediction model. Taking airPLS as an example, the training process and the parameter optimization process of the CNN model were demonstrated. Figure 6A,B shows the optimization process of training loss and the RMSE of the CNN. As the number of training iterations increased, the loss and the RMSE of the training and test sets rapidly decreased close to 0, which meant that the model could fit the training data well, resulting in a significant reduction in the prediction error, and could effectively capture patterns and trends in the data. Figure 6C demonstrates the fitting effect between the predicted values of the CNN model and the actual values. There was a strong correlation between the prediction values of the model and the true values. Additionally, with regard to the evaluation metrics of the model, the R^2^ values of the training set and the test set were 0.9940 and 0.9897, while the RMSECV and the RMSEP were 1.0792% and 1.4045%, respectively. The specific scatter plot of the residuals is shown in Figure 6D. The prediction errors were basically within 3% of the error line. Furthermore, aiming at the raw, S–G + SNV, and Der data, the performance of CNN models trained on these data was also excellent, with the R^2^ values of the test sets being 0.9711, 0.9807, and 0.9886, respectively. The results demonstrated that a handheld near-infrared spectrometer combined with the CNN model could effectively predict the adulteration ratio of *Fritillaria ussuriensis* Maxim. in *Fritillaria cirrhosa* D. Don.

CNNs involve numerous network parameters and require large amounts of data for training, often suffering from slow and time-consuming training processes. To meet the simplicity and speed requirements of handheld NIR spectrometers, this study proposes using PCA for data dimensionality reduction and constructing a CNN model based on the reduced-dimension data. In this research, the first 10 components were utilized for CNN analysis. Figure 6E,F displays the optimized process of training loss and the RMSE of PCA–CNN, both the RMSE and R^2^ dropped rapidly as the number of iterations increased. Figure 6G demonstrates the fitting effect between the predicted values and the actual values of the PCA–CNN model. The R^2^ of the training set and the test set was 0.9945 and 0.9876, and the RMSECV and the RMSEP were 1.0402% and 1.5422%, respectively. Figure 6H shows the residual regression plot, and the prediction error is basically within 3% of the error line. Compared to the full-spectrum data (including preprocessed data), the PCA–CNN model demonstrated comparable performance while significantly reducing modeling computation and iteration time, thus possessing its unique advantages. This indicated that the proposed PCA–CNN could also accurately predict the adulteration ratio of *Fritillaria* spp.

**Figure 7:**
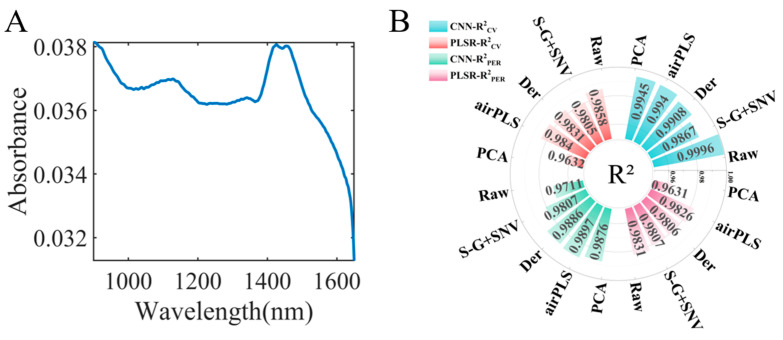
(**A**) Loading information of PC1. (**B**) Summary of regression model results.

It can be seen that the overall performance of the CNN model was superior to PLSR, indicating that the relationship between near-infrared spectroscopy data and adulteration ratios is nonlinear, based on the model results (the summary table of the model performance for adulterated data is shown in Table 1). Therefore, adopting a nonlinear algorithm could yield a more excellent prediction model. Additionally, both PLSR and the CNN could achieve relatively optimal model performance when combined with data after PCA, suggesting that the data after PCA contained a significant amount of information. According to Figure 4D, PC1 captured 100% of the information from samples. Thus, we explored the information on adulteration characteristics by analyzing PC1. Figure 7A shows that the main characteristic peak was concentrated at 1400–1500 nm, indicating that this wavelength range was primarily used to distinguish adulterated *Fritillaria ussuriensis* Maxim. in *Fritillaria cirrhosa* D. Don. Based on our earlier Grad-CAM visualization results (Figure 3B), it can be observed that the main distinguishing areas for *Fritillaria ussuriensis* Maxim. were also concentrated within the 1400–1500 nm range, which aligned with the previous research findings. This validated the effectiveness of both Grad-CAM and the adulterated data model.

## 4. Discussion

In this study, we developed a handheld near-infrared spectroscopy device integrated with a CNN for the dual-quality assessment of *Fritillaria* species: geographical origin authentication and quantification of *Fritillaria ussuriensis* Maxim. adulteration levels in *Fritillaria cirrhosa* D. Don. For the identification of the geographical origin of *Fritillaria* spp., the CNN model achieved an accuracy of 100 ± 0%. Moreover, combined with Grad-CAM, we visualized the feature peaks for each *Fritillaria* species. The visualized information could be verified by references, which demonstrated the validity and interpretability of the proposed strategy. For the prediction of adulteration samples, as shown in Figure 7B, the CNN model also achieved the best performance, with an R^2^ value of 0.9897 for the test set. To meet the demand for simple and rapid detection using handheld near-infrared spectroscopy, we proposed constructing a CNN model after dimensionality reduction using PCA. The R^2^ value of the PCA–CNN model was 0.9876. By analyzing the information of the first principal component (PC1), we found that the primary extracted information was focused on the 1400–1500 nm wavelength range, which was consistent with the visualization region of Grad-CAM. This demonstrated the effectiveness of both Grad-CAM and the adulterated data model.

Based on the R^2^ obtained from the test set, a comparison with other relevant studies is presented below (Table 2). For example, Fan et al. [35] analyzed the adulteration of *Fritillaria cirrhosa* D. Don using NIR. They aimed to identify and quantify *Fritillaria thunbergii* Miq.*, Fritillaria ussuriensis* Maxim.*, Fritillaria pallidiflora* Schrenk*,* Fritillariae hupehensis Bulbus*,* Bulbus Tulipae, and flour. The R^2^ values of the six PLSR models developed were 0.8402, 0.9612, 0.7657, 0.9025, 0.9574, and 0.9761, respectively. Wei et al. [18] quantified the content of *Fritillaria thunbergii* Miq. in *Fritillaria cirrhosa* D. Don using LIBS. The R^2^ value of the PLSR model employed was 0.9983. Jiang et al. [36] utilized ultra-high performance liquid chromatography–triple quadrupole mass spectrometry (UHPLC–QQQ-MS) combined with PLSR modeling for the quantitative analysis of three mixtures: *Fritillaria ussuriensis* Maxim.–*Fritillaria unibracteata*, *Fritillaria thunbergii* Miq.–*Fritillaria unibracteata*, and *F. walujeivii*–*F. delavayi.* The R^2^ values obtained were 0.9949, 0.9721, and 0.9895, respectively. Li et al. [37] employed colorimetric sensor array (CSA) combined with PLSR to quantitatively analyze six samples: *Fritillaria cirrhosa* D. Don and *Fritillaria ussuriensis* Maxim.*, Fritillaria thunbergii* Miq., coix seed (CS), Bulbus Tulipae (BT), magnesium stearate (MGST), and wheat flour (WF). The R^2^ values of the six models were 0.957, 0.905, 0.956, 0.873, 0.923, and 0.906, respectively.

While our approach exhibits slightly lower accuracy compared to the LIBS (R^2^: 0.9983) and UHPLC–QQQ-MS (R^2^: 0.9949) techniques, it offers significant practical advantages in terms of field deployability and operational simplicity. Notably, the proposed method can detect adulterants at a minimum tested concentration of 0.1%. This performance significantly outperforms conventional approaches, such as those listed in Table 2, which are typically limited to detection thresholds ranging from 5% to 25%. This enhanced sensitivity makes our technique a highly effective tool for detecting subtle adulteration in herbal products, particularly in cases involving low-level contamination. By achieving a balance between robust analytical performance (with an R^2^ of 0.9897) and an unprecedented capability to detect low concentrations, the method effectively bridges the gap between laboratory-grade accuracy and on-site applicability. It thus addresses critical challenges in real-world quality control of *Fritillaria* species.

## 5. Conclusions

The results of this study demonstrated that mathematical models for predicting the geographical origin and adulteration content of *Fritillaria* spp. could be accurately established using a handheld near-infrared spectrometer. The proposed visualization strategy exhibited strong chemical interpretability and provided a simple, rapid, and efficient technical means for the quality evaluation of *Fritillaria* spp. These findings highlight the potential of NIR spectroscopy as a valuable tool for ensuring the authenticity and quality of *Fritillaria* spp. in various applications. In practical applications, this methodology could be incorporated into the quality control processes of pharmaceutical companies. During the procurement of *Fritillaria* spp., rapid on-site detection using a handheld near-infrared spectrometer can efficiently determine the origin and detect adulteration. Future research could expand the samples of *Fritillaria* spp. from more geographical regions and different growth stages, which would enhance the generalizability of the models. Furthermore, we could integrate multiple analytical techniques to develop more accurate and reliable quality evaluation systems.

## Figures and Tables

**Table 1:** Summary of model performance for adulterated data. (R^2^ and RMSEC, RMSECV represent the coefficient of determination, the root mean squared error of cross validation, the root mean squared error of prediction, respectively).

Models	Pretreatments	Training		Test	
		R^2^	RMSECV (%)	R^2^	RMSEP (%)
CNN	Raw spectra	0.9996	0.2915	0.9711	2.3524
	PCA	0.9945	1.0402	0.9876	1.5422
	S–G + SNV	0.9867	0.6118	0.9807	1.9201
	Der	0.9908	1.3412	0.9886	1.4789
	airPLS	0.9940	1.0792	0.9897	1.4045
PLSR	Raw spectra	0.9858	1.6622	0.9831	1.7987
	PCA	0.9632	2.6804	0.9631	2.6553
	S–G + SNV	0.9805	1.9525	0.9807	1.9206
	Der	0.9831	1.8173	0.9806	1.9280
	airPLS	0.9840	1.7667	0.9826	1.8226

**Table 2:** Summary of comparison information of similar studies.

Method	Models	Categories of Adulteration	R^2^	Minimum Adulteration Concentration	References
NIR	PLSR	D. Don–Miq.	0.8402	5%	[35]
		D. Don–Maxim.	0.9612		
		D. Don–Schrenk	0.7657		
		D. Don–FHB	0.9025		
		D. Don–BT	0.9574		
		D. Don–flour	0.9761		
LIBS	PLSR-SVR	D. Don–Miq.	0.9983	5%	[18]
UHPLC–QQQ-MS	PLSR	Maxim.–UNI	0.9949	10%	[36]
		Miq.–UNI	0.9721		
		WAL–DEL	0.9895		
CSA	PLSR	D. Don–Maxim.	0.9570	25%	[37]
		D. Don–Miq.	0.9050		
		D. Don–CS	0.9560		
		D. Don–WF	0.8730		
		D. Don–BT	0.9230		
		D. Don–MGST	0.9060		

## Data Availability

The original contributions presented in the study are included in the article/Supplementary Materials, further inquiries can be directed to the corresponding authors.

## Supplementary Materials

The following supporting information can be downloaded at: https://www.mdpi.com/article/10.3390/foods14111907/s1, Figure S1: (A) was average NIR spectra after SG + SNV. (B) was average NIR spectra after airPLS; Table S1: Specific proportion of adulterated samples. Table S2: Architecture of the CNN. Table S3: Summary table of model accuracy results for four algorithms combined with three preprocessing methods. Table S4: The F1 s summary of all models. Table S5: The Pre summary of all models. Table S6: Rec The summary of all models.
