# Supplementary material for: A Dual-Technology Approach: Handheld NIR Spectrometer and CNN for Fritillaria spp. Quality Control
Source: Foods. 2025 May 28;14(11):1907. doi: 10.3390/foods14111907 (PMC12154058; doi:10.3390/foods14111907)
Supplement: Supplementary file 1 [file foods-14-01907-s001.zip › foods-3590976-supplementary.pdf]

Table.S1 Specific proportion of adulterated samples.

**Proportion**					
0.1%	0.5%	1.0%	1.5%	2.0%	2.5%
3.0%	3.5%	4.0%	4.5%	5.0%	5.5%
6.0%	6.5%	7.0%	7.5%	8.0%	8.5%
9.0%	9.5%	10.0%	15.0%	20.0%	25.0%
30.0%	35.0%	40.0%	45.0%	50.0%	

Table.S2 Architecture of the CNN

Layer	Remark	Output size	Normalization Technique	Activation function
Input	750×1 nodes	750×1	—	—
Convolution 1	16 convolutional filters (3 × 1)	750×16	BatchNorm	ReLU
Convolution 2	32 convolutional filters (3 × 1)	375×32	BatchNorm	ReLU
Convolution 3	64 convolutional filters (3 × 1)	187×64	BatchNorm	ReLU
Convolution 4	128 convolutional filters (3 × 1)	94×128	BatchNorm	ReLU
Convolution 5	256 convolutional filters (3 × 1)	47×256	BatchNorm	ReLU
Fully connected layer	32 × 1 nodes	32	—	ReLU
Output	4 × 1 nodes	4	—	Softmax

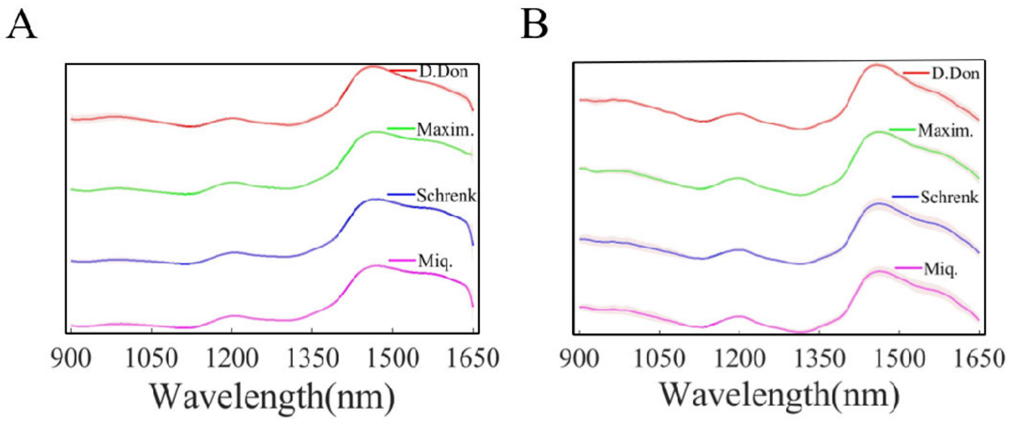

Figure. S1 (A) was average NIR spectra after SG+SNV. (B) was average NIR spectra after airPLS.

Table. S3 Summary table of model accuracy results for four algorithms combined with three preprocessing methods.("Ave" and "Std" denote the mean value and the standard deviation of the accuracy of 20 model cycles, respectively.)

**Models**	**Preprocessing methods**	**Training set**		**Test set**	
		**Ave**	**Std**	**Ave**	**Std**
PLS-DA	Raw spectra	100.00%	0.00%	100.00%	0.00%
	S-G+SNV	100.00%	0.00%	100.00%	0.00%
	Der	100.00%	0.00%	99.92%	0.19%
	airPLS	100.00%	0.00%	100.00%	0.00%
SVM	Raw spectra	85.52%	3.93%	84.02%	4.25%

	S-G+SNV	100.00%	0.00%	100.00%	0.00%
	Der	100.00%	0.00%	100.00%	0.00%
	airPLS	100.00%	0.00%	100.00%	0.00%
DT	Raw spectra	99.67%	0.20%	96.33%	1.48%
	S-G+SNV	100.00%	0.00%	98.63%	0.94%
	Der	100.00%	0.00%	99.50%	0.55%
	airPLS	99.85%	0.17%	97.42%	1.31%
CNN	Raw spectra	100.00%	0.00%	100.00%	0.00%
	S-G+SNV	99.73%	0.89%	99.48%	1.55%
	Der	100.00%	0.00%	100.00%	0.00%
	airPLS	100.00%	0.00%	100.00%	0.00%

Table.S4 The F1 s summary of all models.

Models	Pretreatments	Training		Test	
		**F1 s**	**Std**	**F1 s**	**Std**
PLS-DA	Raw spectra	100.00%	0.00%	100.00%	0.00%
	S-G+SNV	100.00%	0.00%	100.00%	0.00%
	Der	100.00%	0.00%	99.92%	0.18%
	airPLS	100.00%	0.00%	100.00%	0.00%
SVM	Raw spectra	86.21%	3.79%	84.82%	4.08%
	S-G+SNV	100.00%	0.00%	100.00%	0.00%
	Der	100.00%	0.00%	100.00%	0.00%
	airPLS	100.00%	0.00%	100.00%	0.00%
DT	Raw spectra	99.67%	0.19%	96.38%	1.44%
	S-G+SNV	100.00%	0.00%	98.64%	0.73%
	Der	100.00%	0.00%	99.51%	0.54%
	airPLS	99.86%	0.17%	97.44%	1.30%
CNN	Raw spectra	100.00%	0.00%	100.00%	0.00%
	S-G+SNV	99.74%	0.83%	99.52%	1.40%
	Der	100.00%	0.00%	100.00%	0.00%
	airPLS	100.00%	0.00%	100.00%	0.00%

Table.S5 The Pre summary of all models.

Models	Pretreatments	Training		Test	
		**Pre**	**Std**	**Pre**	**Std**
PLS-DA	Raw spectra	100.00%	0.00%	100.00%	0.00%
	S-G+SNV	100.00%	0.00%	100.00%	0.00%
	Der	100.00%	0.00%	99.92%	0.18%
	airPLS	100.00%	0.00%	100.00%	0.00%
SVM	Raw spectra	86.92%	3.65%	85.65%	3.92%
	S-G+SNV	100.00%	0.00%	100.00%	0.00%

	Der	100.00%	0.00%	100.00%	0.00%
	airPLS	100.00%	0.00%	100.00%	0.00%
DT	Raw spectra	99.67%	0.19%	96.43%	1.41%
	S-G+SNV	100.00%	0.00%	98.66%	0.72%
	Der	100.00%	0.00%	99.52%	0.53%
	airPLS	99.86%	0.16%	97.46%	1.28%
CNN	Raw spectra	100.00%	0.00%	100.00%	0.00%
	S-G+SNV	99.76%	0.77%	99.56%	1.25%
	Der	100.00%	0.00%	100.00%	0.00%
	airPLS	100.00%	0.00%	100.00%	0.00%

Table.S6 Rec The summary of all models.

**Models**	**Pretreatments**	**Training**		**Test**	
		**Rec**	**Std**	**Rec**	**Std**
PLS-DA	Raw spectra	100.00%	0.00%	100.00%	0.00%
	S-G+SNV	100.00%	0.00%	100.00%	0.00%
	Der	100.00%	0.00%	99.92%	0.19%
	airPLS	100.00%	0.00%	100.00%	0.00%
SVM	Raw spectra	85.52%	3.93%	84.02%	4.25%
	S-G+SNV	100.00%	0.00%	100.00%	0.00%
	Der	100.00%	0.00%	100.00%	0.00%
	airPLS	100.00%	0.00%	100.00%	0.00%
DT	Raw spectra	99.67%	0.20%	96.33%	1.48%
	S-G+SNV	100.00%	0.00%	98.63%	0.94%
	Der	100.00%	0.00%	99.50%	0.55%
	airPLS	99.85%	0.17%	97.42%	1.31%
CNN	Raw spectra	100.00%	0.00%	100.00%	0.00%
	S-G+SNV	99.73%	0.89%	99.48%	1.55%
	Der	100.00%	0.00%	100.00%	0.00%
	airPLS	100.00%	0.00%	100.00%	0.00%
